# Early linguistic experience shapes bilingual adults’ hearing for phonemes in both languages

**DOI:** 10.1038/s41598-022-08557-7

**Published:** 2022-03-18

**Authors:** Lei Pan, Han Ke, Suzy J. Styles

**Affiliations:** 1 Psychology, School of Social Sciences, Nanyang Technological University, Singapore, Singapore; 2 Centre for Research and Development in Learning (CRADLE), Nanyang Technological University, Singapore, Singapore; 3 Singapore Institute for Clinical Sciences, Agency for Science and Technology Research (A*STAR), Singapore, Singapore

**Keywords:** Language, Perception

## Abstract

English and Mandarin Chinese differ in the voice onset times (VOTs) of /b/ and /p/. Hence the way bilinguals perceive these sounds may show ‘tuning’ to the language-specific acoustic structure of a bilingual’s languages (a discrete model), or a shared representation across languages (a unitary model). We investigated whether an individual’s early childhood exposure influences their model of phoneme perception across languages, in a large sample of early English-Mandarin bilingual adults in Singapore (N = 66). As preregistered, we mapped identification functions on a /b/-/p/ VOT continuum in each language. Bilingual balance was estimated using principal components analysis and entered into GLMMs of phoneme boundary and slope. VOT boundaries were earlier for English than Mandarin, and bilingual balance predicted the slope of the transition between categories across both languages: Those who heard more English from an earlier age showed steeper category boundaries than those who heard more Mandarin, suggesting early bilinguals may transfer their model for how phonemes differ from their earlier/stronger languages to later/weaker languages. We describe the transfer model of discrete phoneme representations and its implications for use of the phoneme identification task in diverse populations.

## Introduction

Most research on language development has tested early language exposure and phonological acquisition in monolingual contexts. Thus far, little is known about the influence of early exposure to more than one language on adult phonological perception. Over the first year of life, infants show an increase in their perceptual abilities for native phoneme perception and a decline for non-native phoneme perception^1–3^, and refinement continues through childhood, for both early and later acquired languages^4–6^. In adults, a hallmark of phoneme perception is higher accuracy in discriminating between similar speech sounds that fall at the boundary between two phoneme categories, but lower accuracy in discriminating between speech sounds when they fall within a single phoneme category. One feature of speech that is often used to investigate categorical perception of phonemes is Voice Onset Time (VOT)—that is, the time span between the release of a stop consonant and the start of vocal fold vibration, as in the English phonemes /b/ and /p/, which differ in their VOT. Most languages have pairs of phonemes that differ in their VOTs^7^, but the nature of the categories can differ between languages. For example, in Russian^8^, /b/ is a prevoiced stop, with a VOT of − 70 ms (i.e., before the release of the stop), and /p/ has near-simultaneous voicing, with a VOT of 18 ms (i.e., after the release of the stop). By comparison, in British English, voiced stops /b, d, g/ have VOTs of around 21 ms, and voiceless stops /p, t, k/, 56 ms^9^. As can be seen in this example, both the timing (earlier VOTs versus later VOTs) and the gap between contrasting phonemes (larger VOT difference versus smaller VOT difference) can vary between languages.

Artificial VOT continua can be used to investigate the nature of phoneme perception in individuals or groups who speak different languages^10–13^. In the phoneme identification task, participants are asked which of the two stimuli is the best match to the auditory word form, and stimuli along a continuum are often treated as belonging to discrete categories, with a steep transition in between (i.e., an ‘s-curve’ identification function). Individuals within one speech community can differ in both the perceptual boundary of their threshold and the steepness of their slope. In the phoneme identification task, children with reading disability show shallower slopes than typically developing children, and illiterate adults show shallower slopes than literate adults^14–16^. Given this relationship between phoneme identification and reading difficulties, some researchers suggest that the phoneme identification task could be useful in identifying children at risk of developing reading difficulties in educational or clinical settings^17^. However, to date all of the key studies of phoneme perception and its relationship to later reading skills have been conducted in monolinguals, potentially limiting the applicability of such tools in bi/multilingual populations.

What do we know about phonological development in bilinguals? In terms of speech production, research in migrant communities has shown that recent migrants transfer the VOTs from their native language into their articulations in their second language, but over time, and across generations their VOT boundaries shift to approximate the VOT norms of the local language environment^18–20^.

In terms of perception, results vary. In one study comparing ten early English-French bilinguals with ten English monolinguals, no significant differences in /d/-/t/ VOT boundaries were observed between groups, but bilinguals showed shallower identification functions than English monolinguals^21^. In a study of eight English–Spanish bilinguals who acquired their second language upon or before entering primary school^22^, response patterns fell into two subgroups: participants who showed an English-like response pattern in both their English and their Spanish tasks (with a steep discrimination function and a boundary close to the monolingual English norm), and participants who showed a bilingual blended or “double standard” pattern, with a shallow discrimination function and a boundary closer to the Spanish norm. Although this study is small it may suggest a unitary model of phoneme perception in bilinguals, with each bilingual showing one pattern of responses in both of their languages. In other samples comparing English/Spanish bilinguals (N = 15) with English monolinguals (N = 15)^23,24^, monolinguals showed a consistent pattern of perception, while bilinguals showed significantly different patterns of perception in their two languages. In these studies, the precise perceptual boundary was associated with self-reported ‘confidence’ scores in understanding, speaking and reading English and Spanish. In recent studies, English native speakers and Mandarin native speakers have shown different perceptual patterns for /b/ and /p/. Zhang^25^ conducted identification tasks to compare the perceptual curves of two English native speakers and two Mandarin native speakers from Mainland China. When hearing tokens drawn from the /b/-/p/ VOT continuum, English monolinguals showed an earlier perceptual boundary (13 ms) than Mandarin monolinguals (36 ms), in line with the category structures of the two languages. However, when testing VOT perception in 10 native Mandarin-speaking late learners of English (learning for 13.5 years on average), the perceptual boundaries of the two test-languages were not significant. This finding suggests that the timing of exposure to different languages may be important in the development of phoneme categories.

The process of ‘tuning’ to the perceptual characteristics of phonemes is known to begin in early childhood. Both English-learning and German-learning infants at 4 months of age can hear a change from /y/ to /u/, but they perform less well in the opposite direction (/u/ to /y/), indicating a pervasive perceptual asymmetry. However, by about 1 year of age, German exposed infants represented /y/ as a discrete category, but English exposed infants do not^26,27^. Human infants therefore start to ‘tune’ their hearing to a language-specific pattern of phoneme perception during their second half of the first year^28,29^. Tuning can involve increases or decreases in sensitivity depending on the languages in question: Between 8 and 10 months of age, Japanese exposed infants show a decline in discriminating between English /r/-/l/, while American English exposed infants show improved discrimination of the same sound pair^2^.

Acoustic categories acquired in early childhood are known to persist into later life, even if exposure ceases. Adult Dutch speakers who were adopted from Korea before the age of 6 months were faster than the Dutch controls in learning and identifying the three-way stop contrast in Korean^4^. Similarly, in Singapore, Hokkien-reared adults who had forgotten their caregiving language were significantly faster in learning Hokkien tones compared to English-reared adults^6^. These ‘lost language’ studies provide powerful evidence that linguistic input in early childhood yields a lasting impact on phonological perception into adulthood.

Taken together, these studies show that early linguistic exposure can create lasting perceptual ‘hangovers’ into adult perception, but outcomes in adult phoneme perception tests may differ depending on the characteristics of the group involved in the study (e.g., early parallel bilinguals, late sequential bilinguals, early exposed ‘lost language’ participants). Among this diversity, no single model of multilingual perceptual development emerges. In all of these studies, bilingualism was treated as a strict grouping variable (monolingual versus bilingual), rather than a complex, multifaceted phenomenon in bilingual populations. Although Garcia-Sierra et al.^23,24^ consider self-reported ‘confidence’ in both languages, their study does not account for within-group differences in the timing or exposure rates of the different languages in the bilingual sample.

In the current study, our primary aim is to establish a model of bi/multilingual perceptual development that may be applied to individuals with a variety of language exposures. For this investigation we therefore focus on individual differences among bilinguals, rather than comparisons between monolinguals and bilinguals. Our target population is a large, diverse sample of early English/Mandarin bilinguals in the highly multilingual context of Singapore. Wu et al.^30^ describes how with bilingualism as a cornerstone of the Singapore education system, and in the 2010 Census, less than 10% of young adults in Singapore reported being literate in only one language (with English and Mandarin the dominant language combination). This population provides access to a large number of bilinguals whose individual patterns of childhood exposure differ (i.e., rates of speech in different languages in the family home), while the languages of exposure remain the same (i.e., Singapore English, Singapore Mandarin).

Thus, the present study examined three questions: (1) Do the perceptual boundaries of bilinguals differ between their languages, in line with the previously documented differences in VOT production? (2) To what extent does early childhood bilingual language exposure influence English and Mandarin VOT boundary locations? (3) To what extent does early childhood bilingual language exposure influence the steepness of a bilingual’s identification function? To conduct a study of individual differences, we recruited a large sample of diverse, early English-Mandarin bilinguals in Singapore.

### Models of English/Chinese bilingual phoneme development

**Figure 1:**
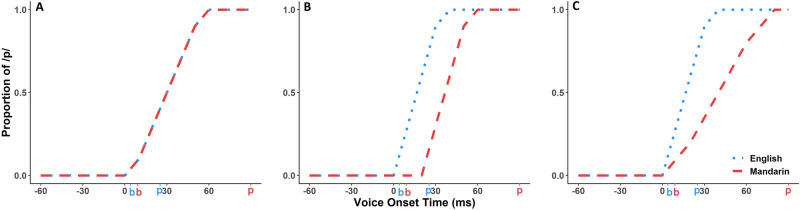
Schematic of three possible models of phoneme perception by bilinguals. Hypothetical identification functions are shown for each of a bilingual’s languages, with prototypical productions for voiced and voiceless stop consonants shown below each model. English = blue; Mandarin = red. (**A**) *Unified model* of phoneme perception with same slope and threshold for perception in both languages. (**B**) *Discrete boundary model* of phoneme perception, with different thresholds for each language. (**C**) *Discrete slope model* of phoneme perception, with different slopes in the identification function for each language.

English and Mandarin Chinese have different category structures across the VOT spectrum. Both languages have a contrastive phoneme pair^31^: The Singapore variety of English typically has relatively early VOT values for stops, with /b, d, g/ and /p, t, k/ at 4 ms and 25 ms, separated by a small VOT difference^32^; The Singapore variety of Mandarin typically has comparatively late VOT values for stops, with /b, d, g/ and /p, t, k/ at 9 ms and 90 ms, separated by a large VOT difference^33^. Since the VOT difference between typical articulations of /b/ versus /p/ is smaller in English than in Mandarin, we can predict different identification functions for the two languages (i.e., a steep transition between 4 and 25 ms in English, and a possible shallower transition between 9 and 90 ms in Mandarin). However, in bilinguals, the representation of speech sounds in these two languages may interact. We propose three theoretical models to describe how bilingual perception may represent the acoustic landscape (see Fig. 1).

#### Unified model

Bilinguals may have one unified pattern of perception whereby they perceive the voicing contrast in the same way across both of their languages. This model approximates the acoustic targets of both languages, as shown by the overlapping identification functions in Fig. 1A.

#### Discrete boundary model

Bilinguals may have a single model for how precisely phoneme categories differ from one another, but they may have different thresholds for the transition in each of their languages. This model accommodates the acoustic targets of each language, as shown by the separate identification functions (earlier, later) in Fig. 1B.

#### Discrete slope model

Bilinguals may have separate models for phoneme category structure in each language, whereby each boundary is adapted to the statistical regularities of the linguistic environment. In this model, if the prototypes of the speech categories are closer together in one language and further apart in the other, the representations may impact the identification function in each language, as shown by the separate slopes (steeper, shallower) in Fig. 1C.

Understanding the nature of phoneme perception and the steepness of the identification function is crucial, as the previous monolingual literature suggests that ‘shallow slopes’ have associations with risk of reading difficulties in children. In the current study, to determine how bilingual speakers resolve phonetic categories in the presence of two linguistic systems that differ in the timing and gap between phonemes on the VOT continuum, we recruited adults who are bilingual in English and Mandarin for this study.

## Methods

We tested phoneme perception in a large sample of early parallel English/Mandarin bilinguals in Singapore, in a phoneme identification task using real words that differ in the voice onset time of the initial consonant. To model phoneme perception in each of the participant’s languages, each participant was tested twice, on different days. To maximize compatibility with other ongoing research, we used a task designed for children. Before preregistering the study and analysis plan, we conducted a pilot study to determine a well powered sample size (see Supplementary Appendix 1, and the OSF repository for this article: https://osf.io/ps9rg/files/).

### Participants

67 early bilinguals in English and Mandarin were recruited for the main study. One participant was excluded and replaced on the basis that they were a late bilingual (monolingual until 10 years old). In total, data was analyzed from 66 participants (age: Median = 21, Range = 19–27; 51 females and 15 males). All participants reported growing up and currently living in Singapore, four participants reported that they had lived a maximum of 3 years out of Singapore. In questions drawn from the Perceptual Experiences Battery Baseline Experimental Screener (PEBBLES)^34^, participants reported ‘normal’ hearing and normal or corrected-to-normal vision, and indicated their day-today hearing in a numeric scale (hearing rating: Median = 7; Range = 2–7). Six participants who gave atypical responses on the hearing screening question were further checked for whether their behavioral responses deviated from the group. All six performed the practice trials normally, indicating they were able to discriminate the auditory test stimuli, and their responses to the main task were well within the range of the group, so no exclusions were made. The study was approved by the Institutional Review Board of Nanyang Technological University (IRB-2019-01-034). Informed consent was obtained from all participants, all of whom were over the age of 19. Experimental procedures were in accordance with the relevant guidelines and regulations.

### Stimuli

Based on the paradigm of acoustic measurements^13^, the stimuli were generated from tokens of natural speech, spliced to create a 16-step VOT continuum ranging from pre-voiced (negative 60 ms) to aspirated (positive 90 ms). Natural speech tokens were drawn from the spoken languages of the local Singapore varieties of both languages. The tokens were produced by a female bilingual native speaker of Singapore English and Singapore Mandarin. Naturalistic speech at a target pitch was elicited using the Pitch Cued Spoken Vowels method^35^. The stimuli were recorded in a sound-attenuating recording booth using a Sennheiser MKH 416 Shotgun Condenser Microphone and a ZOOM H4n Pro Handy Recorder, recording a mono Waveform Audio File Format at a bit depth of 16-bit and a sample rate of 44.1 kHz. The English and Mandarin tokens have different acoustic characteristics that are aligned with the prototypical productions of the target varieties. Amplitude adjustment and noise reduction were applied to all speech tokens in GoldWave Version 6.43. After creating the 16 tokens in the VOT spectrum, the intensity of all tokens was matched to 72.5 dB across tokens for presentation.

### Procedure

**Figure 2:**
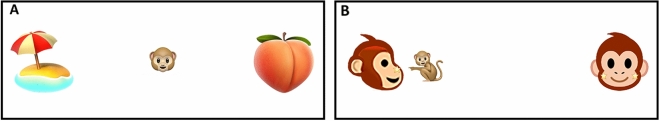
Example screens from the CROWN Game. (**A**) An example stimulus presentation screen of the English phoneme perception task. (**B**) Feedback following choice of target image in the Mandarin phoneme perception task.

We used the English version of the open access task Categorical Recognition for Onsets of Word Names Game (CROWN Game)^36^, and created an adaptation for Mandarin Chinese. Both identification tasks present audio recordings of real words along with easily recognizable pictures. For the English version of the CROWN Game, participants are asked if each speech token is “beach” /biːʧ / or “peach” /piːʧ/ (see Fig. 2A). For the Mandarin version of the CROWN Game, participants are asked if each speech token is “鼻” /biː2/ or “皮” /pʰiː2/, respectively, meaning “nose” and “skin/animal hide” (see Fig. 2B). To motivate participation, the task is embedded in a storyline. In the English version of the task, participants were asked to help a little monkey choose what to do (“go to the beach” or “eat a peach”). In the Mandarin version of the task, they were asked to help the little monkey play a game with the big monkey in which they do one of two actions (“touch the big monkey’s nose” or “touch the big monkey’s skin”). During the game, target images appeared at either side of the screen.

Participants heard a speech token drawn randomly from the VOT continuum, and they were asked to press a key on the corresponding side of the keyboard (“z” for choosing the left image, “/” for choosing the right image) to indicate their decision about which word they heard. After the keypress, a pop sound played and the character was animated to provide audio-visual feedback that the monkey had followed instructions. Participants were invited to take part in both versions of the CROWN Game on two separate days, with the order of the languages randomized. All participants were tested on their own in a sound-attenuating room (one specific testing laboratory in the psychology department). Testing was conducted using a single laptop (Asus Zenbook UX431FL) with a screen resolution of 1366 × 768 and one pair of sound-attenuating, encapsulating headphones (Audio-Technica ATH-M40x Professional Studio Monitor Headphones). At the beginning of the task the output volume of the laptop was set to 50% using the laptop’s operating system, and participants were instructed not to change the volume. Instruction languages were yoked to the test-language of the task for that day. After the instructions, there were two practice blocks in which participants were asked to choose the matching picture in response to tokens of /b/ and /p/ drawn from the ends of the VOT continuum (four practice trials in each block). Each experimental block contained one presentation of each of the 16 tokens in the VOT continuum, repeated for a total of ten blocks. The position of the visual stimuli was swapped at the beginning of each new block. After the two separate sessions, participants were asked to fill in demographic information, and a Language Fingerprint survey.

### Measurements

Generalized Linear Mixed-Effects Models (GLMMs) were conducted to test the effects of language balance on phoneme perception of bilinguals in their two languages. The following variables were computed for data analysis.

#### Early language exposure

In the Language Fingerprint survey, participants provided the proportion of English, Mandarin and other languages/dialects spoken by the people they grew up with, and they specified the amount of time each caregiver spent talking to them. To quantify the amount of exposure to both target languages during their early childhood, a Composite Language Input Profile^37^ was computed for English (CLIP-E) and for Mandarin (CLIP-M).

#### Age of acquisition

Participants reported the age at which their caregivers started using English/Mandarin with them. Age of acquisition of English (AoA-E) and age of acquisition of Mandarin (AoA-M) were collected by using the Language Fingerprint survey.

#### VOT boundary and slope

The crossover point is the point on the VOT continuum at which an individual switches from making majority /b/ decisions to making majority /p/ decisions (i.e., at the point where 50% of decisions are /b/ and 50% of decision are /p/), which is defined as the VOT boundary. The steepness of the identification function is the slope value at the crossover point, representing how steep the transition is from one phoneme category to another. Individual responses were measured as the proportion of /p/ choices allowing a two-parameter psychometric function to be fitted by using the quickpsy R package^38^. The fitted psychometric curve was defined in part by the 50% crossover point and the slope of the curve at midpoint threshold. VOT boundary and slope were automatically computed after the curve fitting. The curve fitting was performed separately for each language.

Following a small pilot study and power analyses, we preregistered the new analysis plan and stop rule before beginning the main study.

## Results

### Language exposure pattern

Participants answered a series of questions about their backgrounds in the Language Fingerprint survey. Some questions were used as screening questions to check participants are sufficiently familiar with the Singapore variety of English and the Singapore variety of Mandarin, with limited residency outside Singapore, and that they identify as early bi/multilinguals of English and Mandarin. Some questions in the Language Fingerprint survey help to characterize the nature of bilingual exposure in the group. Table 1 shows summary data from the Language Fingerprint survey where it is clear that our participants were exposed to substantial amounts of both English and Mandarin by the age of 5 years, and all reported fluent use of both languages in a variety of contexts by the age of 8 years. Full language background data can be found in the OSF repository for this project (https://doi.org/10.17605/OSF.IO/PS9RG).

**Table 1:** Summary of language background data.

Language backgrounds	Minimum	Maximum	Median
Age of acquisition^a^: English (years old)	0	9	0
Age of acquisition^a^: Mandarin (years old)	0	6.3	0
Self-reported hearing rating	2	7	7
Self-reported proficiency: understanding English	5	7	7
Self-reported proficiency: understanding Mandarin	3	7	6
Self-reported proficiency: speaking English	5	7	7
Self-reported proficiency: speaking Mandarin	3	7	5
Years spent in a family where English is spoken	11	25	20
Years spent in a family where Mandarin is spoken	3	27	20
Self-reported age of fluent use of English (years old)	1	13	5
Self-reported age of fluent use of Mandarin (years old)	2	15	5
Age first studied English at school (years old)	2	9	4
Age first studied Mandarin at school (years old)	2	9	5
Years resident outside Singapore	0	3	0
Years resident in a country where English is spoken	15	27	20.5
Years resident in a country where Mandarin is spoken	2	27	20.5

^a^Age of Acquisition: “Age when your caregivers started using this language with you”. In cases where question was misinterpreted, youngest age adjusted to 0.

Participants self-rated the proficiency of their languages in the domains of ‘understanding when people speak’ and ‘speaking to others’ on a seven-point scale (7 = native-level, 1 = a few words, 0 = I don’t understand/speak this language). As shown in Table 1, all participants reported strong language skills in English (Understanding: Median = 7, Range = 5–7; Speaking: Median = 7, Range = 5–7), and a range of language skills in Mandarin (Understanding: Median = 6, Range = 3–7; Speaking: Median = 5, Range = 3–7).

The core language exposure variables in the preregistered study design are age of acquisition in each language and the proportion of early language input in each language, as measured by the CLIP. An overview of participant diversity can be seen in the archive of this project (https://osf.io/3nwud/). Computed CLIPs ranged from 0 to 98% exposure to English in early childhood (Median = 60.3), and 0% to 100% exposure to Mandarin in early childhood (Median = 35.9).

Table 2 shows correlations among target language background variables. To reduce multicollinearity for analysis by GLMM, we conducted a PCA to extract the factors that can best explain the total shared variance. The Kaiser–Meyer–Olkin measure of sampling adequacy (KMO = 0.717) and the Bartlett's test of sphericity (χ^2^(6) = 213.28, *p* < 0.001) suggested that a factor analysis was appropriate for the correlated variables. The results of the factor analysis are shown in Table 2. Factors explaining more than 25% of the total variance were considered as principal components, resulting in a single significant factor termed ‘bilingual balance’. Secondary analysis with two extracted components led to the same pattern of results (see Supplementary Table 5). CLIP-E, CLIP-M and AoA-E had large component loadings on bilingual balance, such that an individual with a high factor score had more early English input, less early Mandarin input, and acquired English earlier than an individual with a low factor score.

**Table 2:** Descriptive statistics, Pearson correlation matrix, component loadings and communalities from a Principal Component Analysis for independent variables.

Variables	1	2	3	4	Mean	SD	Component loadings	Communalities
1. AoA_English_	1.00				1.01	2.12	0.83	0.68
2. AoA_Mandarin_	− 0.23	1.00			0.69	1.44	− 0.53	0.28
3. CLIP_English_	− 0.72**	0.39**	1.00		56.85	25.45	− 0.96	0.92
4. CLIP_Mandarin_	0.70**	− 0.40**	− 0.96**	1.00	38.95	26.51	0.95	0.91

One component (the bilingual balance factor) was extracted with an initial eigenvalue of 2.79.

** indicates correlation is significant at the alpha level of 0.01 (2-tailed).

### Behavioral response and curve fitting

**Figure 3:**
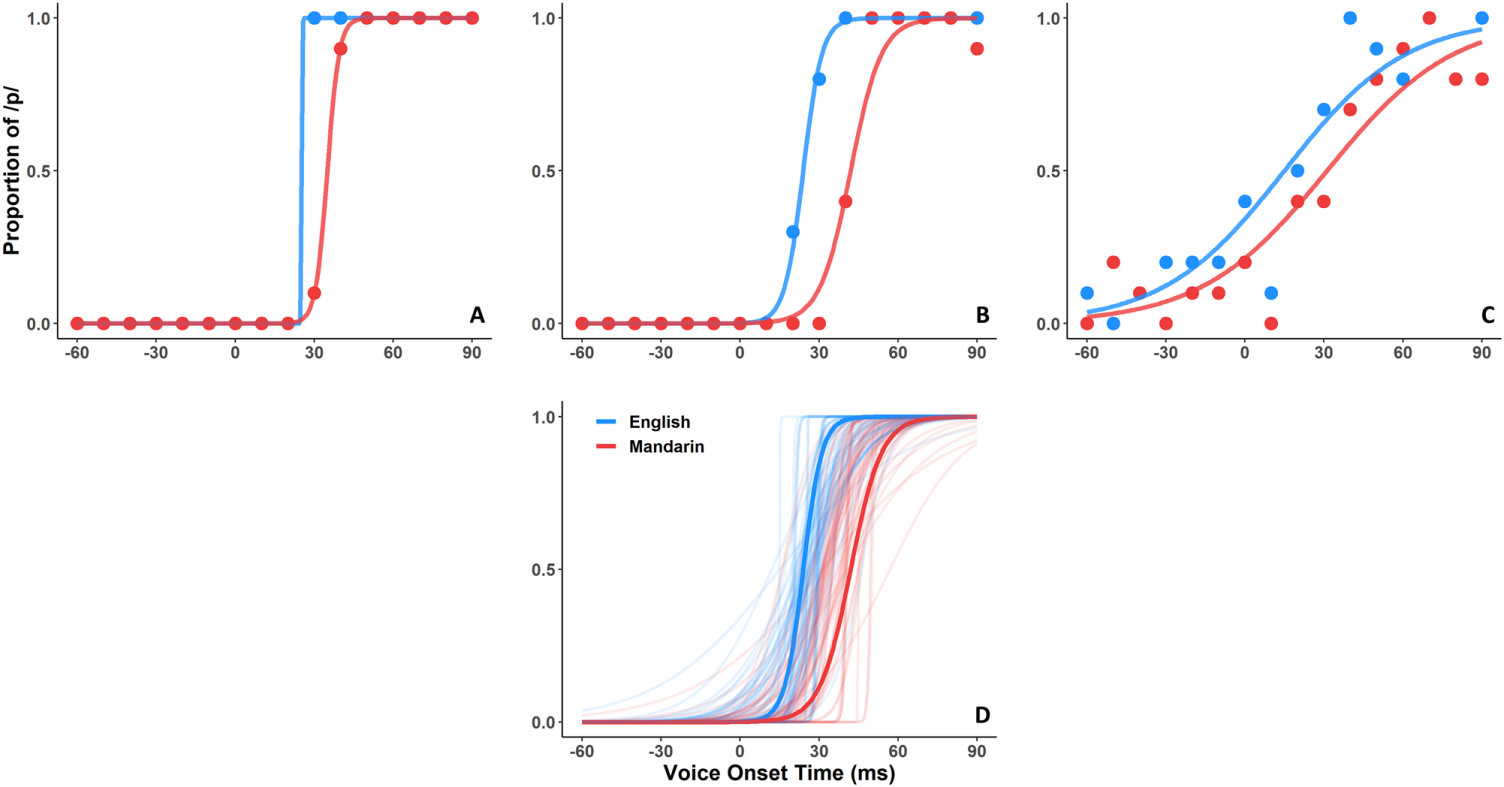
Psychometric curve fitting for three individuals with highest (**A**), median (**B**) and lowest slope values (**C**). Psychometric curves for all 66 participants, with median participant highlighted (**D**). Each language shown separately.

Each participant made 10 decisions on each of 16 VOT steps, allowing a psychometric function to be fitted. Figure 3 shows examples of the curve fitting procedure, and the psychometric functions of all participants with median highlighted (Fig. 3D). Full curve fitting for all participants is shown in Supplementary Fig. 2.

### VOT boundary

**Figure 4:**
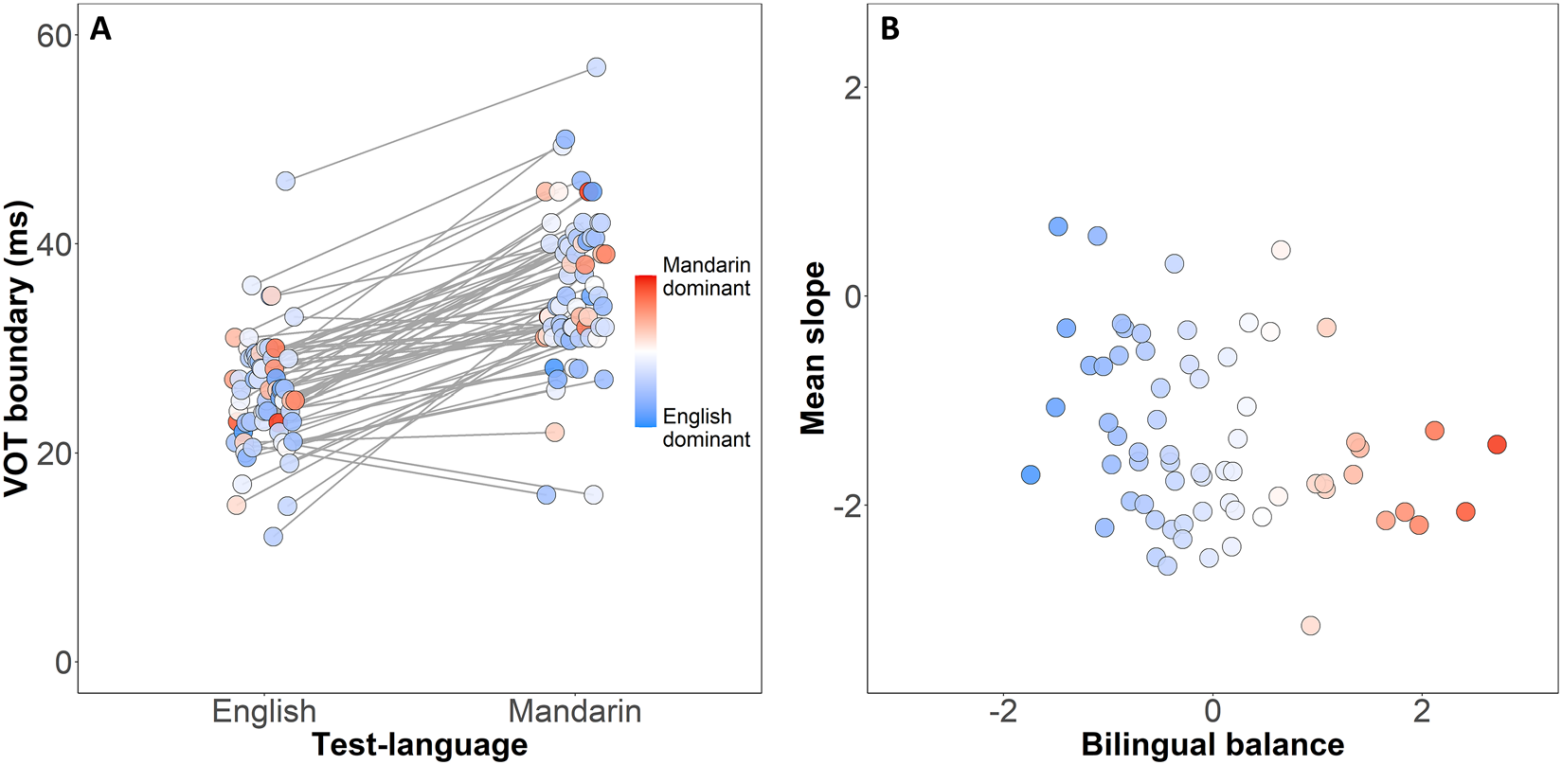
Results of GLMMs on VOT boundary and slope showing significant effects. (**A**) VOT boundaries for each participant across two languages. (**B**) Scatter plot of slope values (pooled across languages) with bilingual balance derived from PCA. Early bilingual balance shown in a color scale.

The VOT boundary between the two bilabial stops is estimated as the 50% crossover point of the psychometric function for an individual participant. Figure 4A shows the location of VOT boundary for each individual in each task language, with the PCA derived bilingual balance factor scaled from blue (the English dominant end of the scale) to red (the Mandarin dominant end of the scale). Note that the scale represents relative differences within the group, so the midpoint of the scale reflects a typical exposure pattern.

As outlined in the preregistration, a GLMM was implemented by fitting the lmer function in the lme4 R package^39^ with two test-languages sum coded as − 0.5 and 0.5. The test-language of the phoneme perception task, and the bilingual balance factor derived in the PCA were incorporated as fixed effects, along with the interaction, with by-participant random intercepts. As shown in Table 3 (Left), Test-language had a robust effect on VOT boundary (*SE* = 0.76, *t* = 13.00, *p* < 0.0001). In line with the known VOTs of typical productions of English and Mandarin in the local context, phoneme boundaries typically occurred at earlier VOTs in the English language test (*M*_English_ = 26 ms, *SD* = 5.3) than in the Mandarin language test (*M*_Mandarin_ = 36 ms, *SD* = 7.2). There were no further significant effects or interactions. Hence, we observed discrete VOT boundaries for bilingual speakers of English and Mandarin, without significant influence from each speaker’s unique pattern of early childhood language exposure.

**Table 3:** Fixed effects in linear mixed-effect model including fixed effects of test-language and bilingual balance, and random effects of participants (random intercept) for VOT boundary (Left) and slope (Right).

	VOT boundary (ms)				Slope			
	Estimate (95% Conf. Int)	SE	*t* value	*p* value	Estimate (95% Conf. Int)	SE	*t* value	*p* value
**Fixed effects**								
(Intercept)	30.68 (29.33 to 32.02)	0.68	45.28	** < 0.0001**	− 1.37 (− 1.57 to − 1.18)	0.10	− 13.94	** < 0.0001**
Test-language (Mandarin)	9.87 (8.36 to 11.38)	0.76	13.00	** < 0.0001**	− 0.24 (− 0.55 to 0.08)	0.16	− 1.50	0.1386
Bilingual balance	0.23 (− 1.12 to 1.59)	0.68	0.34	0.732	− 0.24 (− 0.44 to − 0.04)	0.10	− 2.42	**0.0183**
Interaction	− 0.01 (− 1.53 to 1.51)	0.76	− 0.01	0.990	0.12 (− 0.19 to 0.44)	0.16	0.78	0.4407
**Random effects**								
Residual	19.02 (SD = 4.36)				0.82 (SD = 0.91)			
By-participant intercept	20.79 (SD = 4.56)				0.23 (SD = 0.48)			
N	66				66			
Observations	132				132			
AIC/BIC	851.9/869.2				389.8/407.1			
log-likelihood	− 419.9				− 188.9			

Larger t value in the GLMM for VOT boundary indicates a later threshold (ms) in the VOT continuum.

Smaller t value in the GLMM for slope value indicates a shallower slope of the phoneme identification curve.

Significant values are in bold.

### Slope

The steepness of the slope at the transition between the two bilabial stops is estimated at the 50% crossover point of the psychometric function for an individual participant. As outlined in the preregistration, to find out whether the effects observed in the pilot study would replicate in a large, well powered sample, a GLMM was implemented by fitting the lmer function^39^. The test-language of the phoneme perception task and the bilingual balance factor derived in the PCA were incorporated as fixed effects, along with the interaction. Two levels of test-language (English and Mandarin) were sum coded as − 0.5, 0.5. By-participant intercept was incorporated as a random effect in the model. As shown in Table 3 (Right), test-language did not have a significant impact on the slope value (*SE* = 0.16, *t* =  − 1.50, *p* = 0.1386). However, bilingual balance was a strong predictor of the overall steepness of the fitted identification curve for an individual (*SE* = 0.10, *t* =  − 2.42, *p* = 0.0183), with steeper slopes for people whose bilingual balance was closer to the English dominant end of the scale, as shown in Fig. 4B. No significant interactions were found between the two effects. Hence, we observed that each participant had a general slope for their perception in both languages, but slope was steeper for those participants who were more dominant in English than for participants at the other end of the bilingual balance scale.

## Discussion

Many previous studies of bilingualism and perception of speech sounds have relied on small-scale comparisons between monolinguals and a group of bilinguals who are treated as homogeneous. In the current study, our sample of bilinguals was more than three times larger than previous studies of this kind, which allowed us to treat early childhood bilingual experience as a continuous variable rather than a simple grouping variable. This allows us to focus on how bilinguals differ among themselves, rather than focusing on how bilinguals (as a group) differ from monolinguals.

We observed that bilinguals in our sample typically showed a difference in the way they perceive VOT boundaries for English and in Mandarin (earlier VOT boundary for English, later VOT boundary for Mandarin). This difference is aligned with previously documented production data for the local varieties of these languages, and the difference was not influenced by early childhood language exposure. However, a participants’ score on the bilingual balance scale (derived from their self-reported language exposure patterns in early childhood) predicted the shape of their overall phoneme identity function (shallower for more/earlier Mandarin exposure, steeper for more/earlier English exposure). These observations allow us to refine our models of possible bilingual phoneme perception.

**Figure 5:**
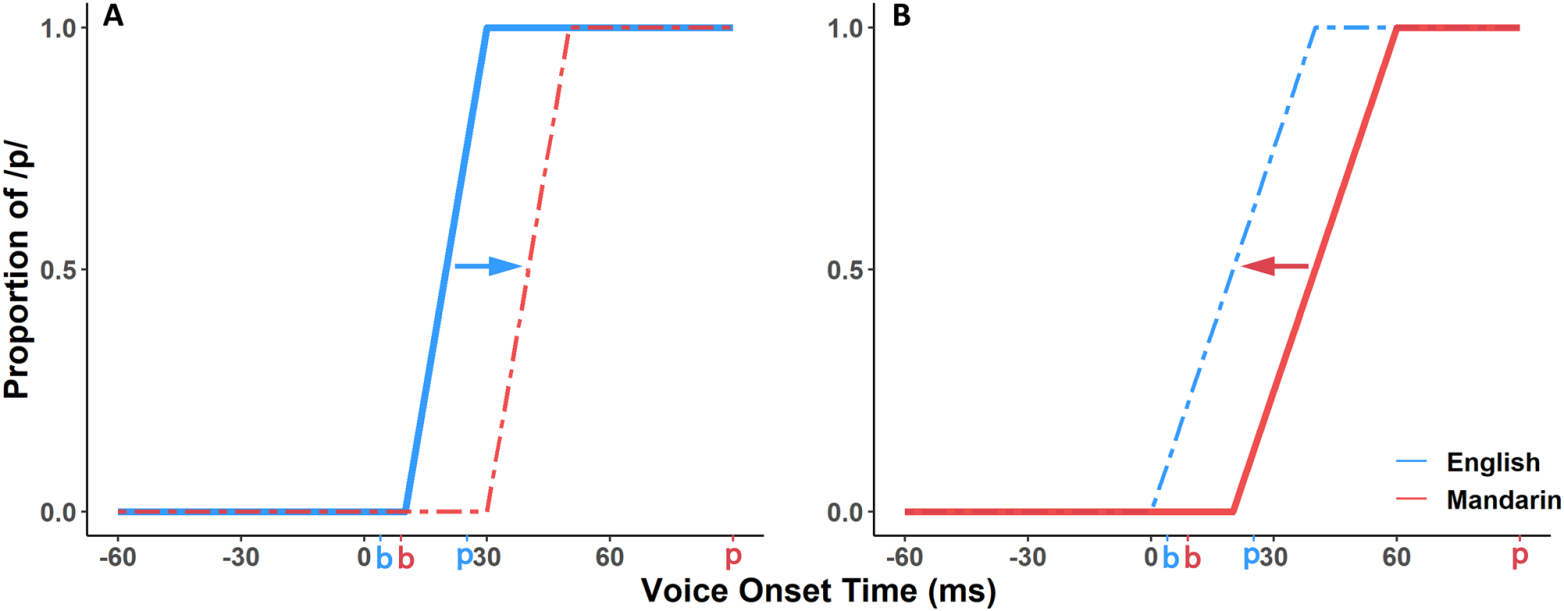
Schematic of the *Slope transfer model* of phoneme perception for bilinguals. Prototypical productions for voiced and voiceless stop consonants are shown. English = blue; Mandarin = red. (**A**) A hypothetical English-dominant bilingual with English-tuned steep slope transferred to the category boundary position for perception of later acquired Mandarin. (**B**) A hypothetical Mandarin-dominant bilingual with Mandarin-tuned slope transferred to the category boundary position for perception of later acquired English.

The finding of discrete representations for VOTs of English and Mandarin partially support a *Discrete boundary model* of speech perception for bilinguals, with earlier perceptual thresholds for the language with earlier prototypical pronunciations. However, in both the small-scale pilot and the full-scale study, the correlation between bilingual balance and the slope of the identification deviates from the predictions of the discrete boundary model. The finding of a shared slope differing by language exposure was unexpected, and did not conform to any of our prior models of bilingual phoneme perception. One possible explanation is that a child might ‘tune’ their VOT perception to one language in early childhood, and then transfer their perceptual model to a weaker or later acquired language by shifting the perceptual boundary to an earlier or later VOT, but not by changing the characteristics of the slope. Figure 5 is a schematic of how a child might tune their acoustic sensitivity to the statistical structure of one language, and then transfer that model of phoneme differences to a new boundary location for another language. We call this the *Slope transfer model* of bilingual phoneme perception. In the current paper we observe Slope transfer in combination with the *Discrete boundary model*.

‘Tuning’ to the perceptual categories of first languages has been widely observed^2,26–29^, and the perceptual effects of this early exposure are evident even for languages that are later ‘lost’^4,6^. However, some aspects of tuning continue throughout childhood. One study on phoneme perception of Mandarin stops suggested that the discrimination abilities of VOTs were still in development among adolescents aged 12 to 14 years^40^. A study on phoneme perception of English /b/ and /p/ in 6-year-old English/Chinese bilingual children from Singapore^41^, also showed a clear decision gradient along a VOT spectrum, but lower slope values than adults in the current study suggest that phoneme perception is still developing.

While the current study reveals that early bilingual balance influences the slope of phoneme perception of bilingual adults, the aforementioned children exhibited substantial variability in slope values, but the variability was independent from their language exposure^41^. A possible explanation for the differences in findings from the same linguistic community is that the patterns of perception by bilinguals may become stronger with age, due to years of use; The more time bilinguals spend using their childhood languages, the more strongly their perceptual pattern emerges. Alternatively, lower slope values in children’s responses may indicate more noise in their pattern of responses, and hence a low power to detect a true effect.

Investigation of differences among bilinguals can be complex given the diversity of language exposure patterns, and this challenge is compounded when sample sizes are small. To account for this challenge, we designed a study specifically to investigate within-group differences, using a novel combination of language self-reporting tools, and big-data analytic methods to characterize bilingualism within the diverse group. The self-reporting tools include questions about age of acquisition of different languages, and rates of exposure from different caregivers in early childhood, providing a nuanced view of bi/multilingualism. Despite the complex, multidimensional structure of each person’s unique ‘language fingerprint’, the use of data-reduction techniques allows us to identify sources of shared variance among the pattern of responses, thereby creating a population-relevant index of ‘bilingual balance’. To ensure sufficient sample size for a within-group analysis we conducted a pilot study, and used the measured effect size to preregister a well-powered study. This novel combination of sociolinguistic self-reporting tools, computational techniques, and robust research design brings new insights into relationships between early language exposure and perception into adulthood.

In terms of the study’s predictions about models of bilingual phoneme perception, the findings support discrete phoneme representations in bilinguals’ two languages, but we describe a novel pattern of phoneme structure ‘transfer’ from a language that was more strongly represented in early childhood to a language that was less strongly represented in early childhood. According to this ‘transfer’ model of bilingual phoneme perception, those participants who had more early childhood exposure to the language with the smaller VOT difference between prototypical pronunciations of /b/ and /p/ (here, English) develop a steeper slope for phoneme perception of VOT, which they then apply to the perceptual boundary for their other language (here, Mandarin Chinese). While this pattern is optimally tuned for English, it would also adequately capture the phoneme contrast in Mandarin, as the transition part of the psychometric function falls between the Mandarin prototypes. By contrast, those participants who have more early childhood exposure to the language with the larger VOT difference between prototypical pronunciations of /b/ and /p/ (here, Mandarin Chinese) develop shallower slopes, which they also apply to their other language (here, English). In this case, although the slope is optimally tuned to the wide gap between Mandarin prototypes, the sloping part of the psychometric function extends beyond both English language prototypes. This may mean that speakers with this profile experience more ambiguities when listening to spoken English due to the fact that many of the tokens fall in the region of perception where the person may be less certain whether the sound should be classified as a /b/ or a /p/. Speakers with this profile may therefore weight linguistic cues differently, placing more weight on semantic context relative to strictly acoustic cues. To the best of our knowledge, this is the first report of exposure-dependent transfer of a category identification function from one language to another, and the first report of asymmetries driven by the acoustic difference between phoneme prototypes in two languages that share a phoneme contrast, but differ in the structure of their phoneme categories.

## Conclusions

This study provides clear evidence of discrete phonological categories in bilinguals, and early bilingual balance influencing the overall slopes of the identification function for both languages. As an individual’s slope in a phoneme identification task is known to predict reading difficulties in children^14,15^, some researchers suggests that phoneme identity tests may be valuable in screening programmes designed to identify children at risk of reading difficulties^17^. Our findings suggest that this kind of test should be treated with caution in bilinguals due to possible transfer of slope values from early acquired languages. Understanding the characteristics of phoneme perception across the lifespan in bilinguals is crucial for evaluating the validity of such tests in global learning environments. Furthermore, we demonstrate the value of taking an individual differences approach to patterns of perception among bilinguals rather than focusing on differences group-level differences between bilinguals and monolinguals.

## Supplementary Information

Supplementary Information.

## Data Availability

Data, Code, Stimuli and Supplementary Materials can be found on the OSF repository for this project (Pan, Ke & Styles, 2022: https://doi.org/10.17605/OSF.IO/PS9RG). Preregistration was archived on OSF on August 6, 2020 (Pan, Ke & Styles, 2020: https://doi.org/10.17605/OSF.IO/4Y6CB).
