# Supplementary material for: Early linguistic experience shapes bilingual adults’ hearing for phonemes in both languages
Source: Sci Rep. 2022 Mar 18;12:4703. doi: 10.1038/s41598-022-08557-7 (PMC8933432; doi:10.1038/s41598-022-08557-7)
Supplement: Supplementary file 1 — Supplementary Information. [file 41598_2022_8557_MOESM1_ESM.pdf]

Early linguistic experience shapes bilingual adults' hearing for phonemes in both languages

Lei Pan, Han Ke & Suzy J Styles

Supplementary Appendix 1.

To detect the effect of early linguistic experience on categorical perception of VOTs, we first conducted a pilot study and analyzed the data in line with a draft pre-registration. 15 bilingual English-Mandarin adults (age: M = 21.0, SD = 1.71, age range 19-25; 12 females and 3 males) with normal or corrected-to-normal vision and hearing took part in the pilot study. All participants reported growing up and currently living in Singapore.

GLMMs incorporating fixed effects of test-language, proportion of early English input, AoA-E, AoA-M and random effects of participant (random intercept) were implemented on each of the dependent variables of interest (VOT threshold and slope) by fitting the lmer function in the lme4 R package. In the GLMM of VOT boundaries, the language of test showed a significant effect on the position of an individual's VOT threshold ($\beta = 20.25$ (ms), $t = 3.99$, $p = 0.0012$, $|f^2| = 0.40$). As indicated in Supplementary Figure 1, VOT boundaries for English are significantly earlier than for Mandarin. By contrast, in the GLMM for slope values, no language-specific differences were observed, however, there was a significant effect of ratio of early language exposure on the slope of the identification function in this small sample ($\beta = 1.79$, $t = 2.82$, $p = 0.0127$, $|f^2| = 0.31$), such that slopes for participants with more early exposure to Chinese have lower slopes overall.

These findings suggest that bilinguals in our pilot sample have different thresholds for VOT perception in their two languages. Contrary to our predictions, the relative rate of exposure to English versus Mandarin influenced the overall steepness of a participant's general identification curve strategy across both of their languages, as though the slope for the early language was mapped onto the later language, as an adaptation to refine the position of the threshold.

On the basis of the pilot study, a power analysis was conducted to determine the required sample size for a pre-registered main study. Estimated sample size is calculated by G*power Version 3.1.9.4 using the effect sizes observed in the pilot study as a benchmark. Different effect sizes were obtained from the models and we chose the smaller one as the estimate in the power analysis to generate a larger required sample size. To achieve a power of 0.95 with an effect size ($|f^2|$) of 0.31 at and alpha-level of 0.05, we determined a total sample size of 66 would be

required in the main experiment if the effect size was at least the same size as in the pilot. In the main study, we planned to run a Principal Component Analysis to include all linguistic background variables in a multifactorial model of bilingualism, while avoiding multicollinearity in analysis. As the sample size of the pilot was not large enough to meet the meet the minimum requirement of an exploratory factor analysis, we used the ratio of early language exposure as a proxy for more-nuanced relationships between language factors.

Supplementary Figure 1.

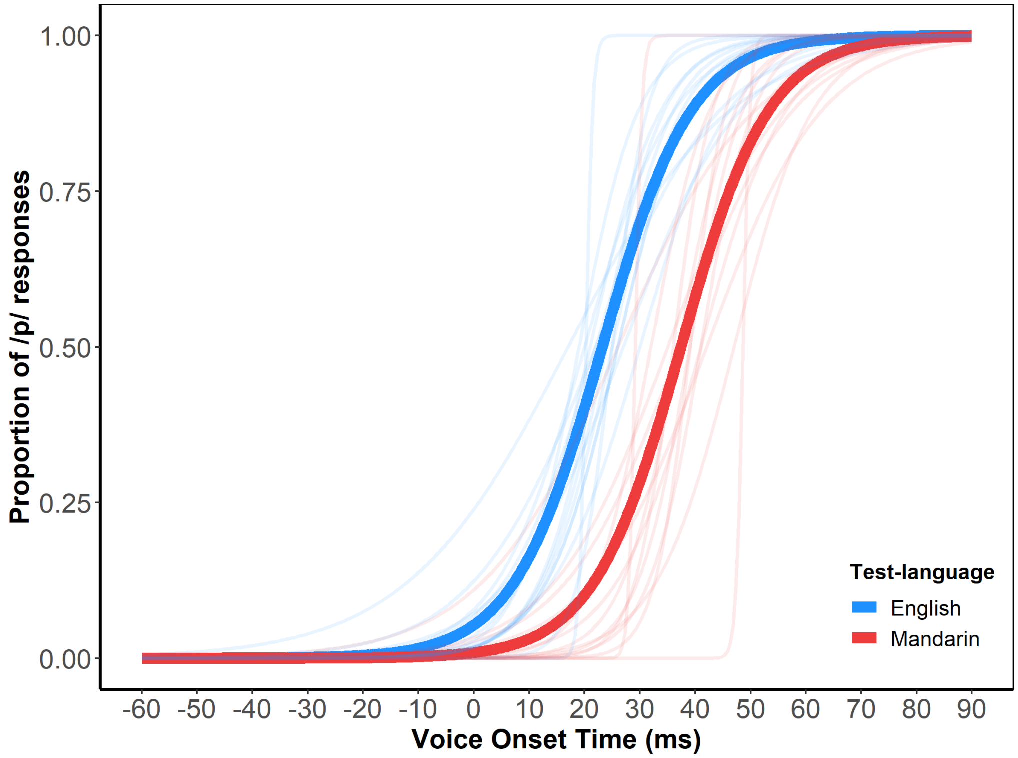

Fitted psychometric curves for each participant with the average slope for English and the average slope for Mandarin phoneme identification task highlighted (pilot study: N = 15).

Supplementary Table 1.

Fixed effects in linear mixed-effect model for VOT boundary (ms) including fixed effects of test-language and linguistic experience, and random effects of participants (random intercept) in the pilot study (N = 15).

**Fixed effects**	**Estimate**	**Std. Error**	***t* value**	***p* value**
(Intercept)	29.12	4.43	6.58	**<0.0001**
1. Test-language (Mandarin)	20.25	5.08	3.99	**0.0012**
2. Early input ratio (English)	-6.57	6.05	-1.09	0.2873
3. AoA-E	-1.45	1.37	-1.06	0.2994
4. AoA-M	-17.03	8.14	-2.09	**0.0460**
1:2	-9.98	6.94	-1.44	0.1714
1:3	-0.10	1.57	-0.64	0.5351
1:4	13.46	9.35	1.44	0.1706
2:3	-0.35	2.34	-0.15	0.8825
2:4	18.09	8.92	2.03	0.0526
3:4	3.65	1.65	2.21	**0.0361**
1:2:3	4.58	2.68	1.71	0.1086
1:2:4	-13.22	10.25	-1.29	0.2166
1:3:4	-3.67	1.90	-1.93	1.0726
2:3:4	-3.22	1.63	-1.98	0.0581
1:2:3:4	4.09	1.87	2.19	**0.0451**
**Random effects**				
Residual	5.25 (SD = 2.29)			
By-participant intercept	2.72 (SD = 1.65)			
N	15			
Observations	30			
AIC / BIC	181.5 / 206.8			
logLikelihood	-72.8			

Larger t value in the GLMM for VOT boundary indicates a later crossover point (ms) in the VOT continuum.

Effect size (f^2) of the test-language on VOT boundary in the model is 0.4025245.

Supplementary Table 2.

Reproducibility analysis for the pilot study (N = 15), based on the effect of test-language on VOT boundary.

**F tests** – Linear multiple regression: Fixed model, R^2 increase			
**Analysis:**	A priori: Compute required sample size		
**Input:**	Effect size f^2	=	0.4025245
	α err prob	=	0.05
	Power (1−β err prob)	=	0.95
	Number of tested predictors	=	4
	Total number of predictors	=	4
**Output:**	Noncentrality parameter λ	=	20.9312740
	Critical F	=	2.5695400
	Numerator df	=	4
	Denominator df	=	47
	Total sample size	=	52
	Actual power	=	0.9537401

Supplementary Table 3.

Fixed effects in linear mixed-effect model for slope value including fixed effects of test-language and linguistic experience, and random effects of participants (random intercept) in the pilot study (N = 15).

**Fixed effects**	**Estimate**	**Std. Error**	***t* value**	***p* value**
(Intercept)	-0.92	0.46	-1.98	0.0658
1. Test-language (Mandarin)	0.17	0.08	2.09	0.0545
2. Early input ratio (English)	1.79	0.63	2.82	**0.0127**
3. AoA-E	0.32	0.14	2.24	**0.0406**
4. AoA-M	0.256	0.85	0.30	0.7686
1:2	-0.17	0.11	-1.55	0.1431
1:3	-0.07	0.02	-2.83	**0.0128**
1:4	-0.27	0.15	-1.85	0.0836
2:3	-0.51	0.24	-2.09	0.0540
2:4	-0.49	0.94	-0.52	0.6104
3:4	-0.08	0.17	-0.46	0.6550
1:2:3	0.07	0.04	1.64	0.1224
1:2:4	0.29	0.14	1.83	0.0867
1:3:4	0.04	0.03	1.22	0.2421
2:3:4	0.14	0.17	0.84	0.4157
1:2:3:4	0.11	0.03	3.74	**0.0020**
**Random effects**				
Residual	0.001 (SD = 0.04)			
By-participant intercept	0.086 (SD = 0.29)			
N	15			
Observations	30			
AIC / BIC	-4.9 / 20.4			
logLikelihood	20.4			

Smaller t value in the GLMM for slope value indicates a shallower slope of the phoneme identification curve. Effect size (f^2) of the early language input on slope in the model is 0.3071895.

Supplementary Table 4.

Reproducibility analysis for the pilot study (N = 15), based on the effect of early language input on slope.

**F tests** – Linear multiple regression: Fixed model, R^2 increase			
**Analysis:**	A priori: Compute required sample size		
**Input:**	Effect size f^2	=	0.3071895
	α err prob	=	0.05
	Power (1–β err prob)	=	0.95
	Number of tested predictors	=	4
	Total number of predictors	=	4
**Output:**	Noncentrality parameter λ	=	20.2745070
	Critical F	=	2.5226149
	Numerator df	=	4
	Denominator df	=	61
	Total sample size	=	66
	Actual power	=	0.9520156

Supplementary Figure 2.

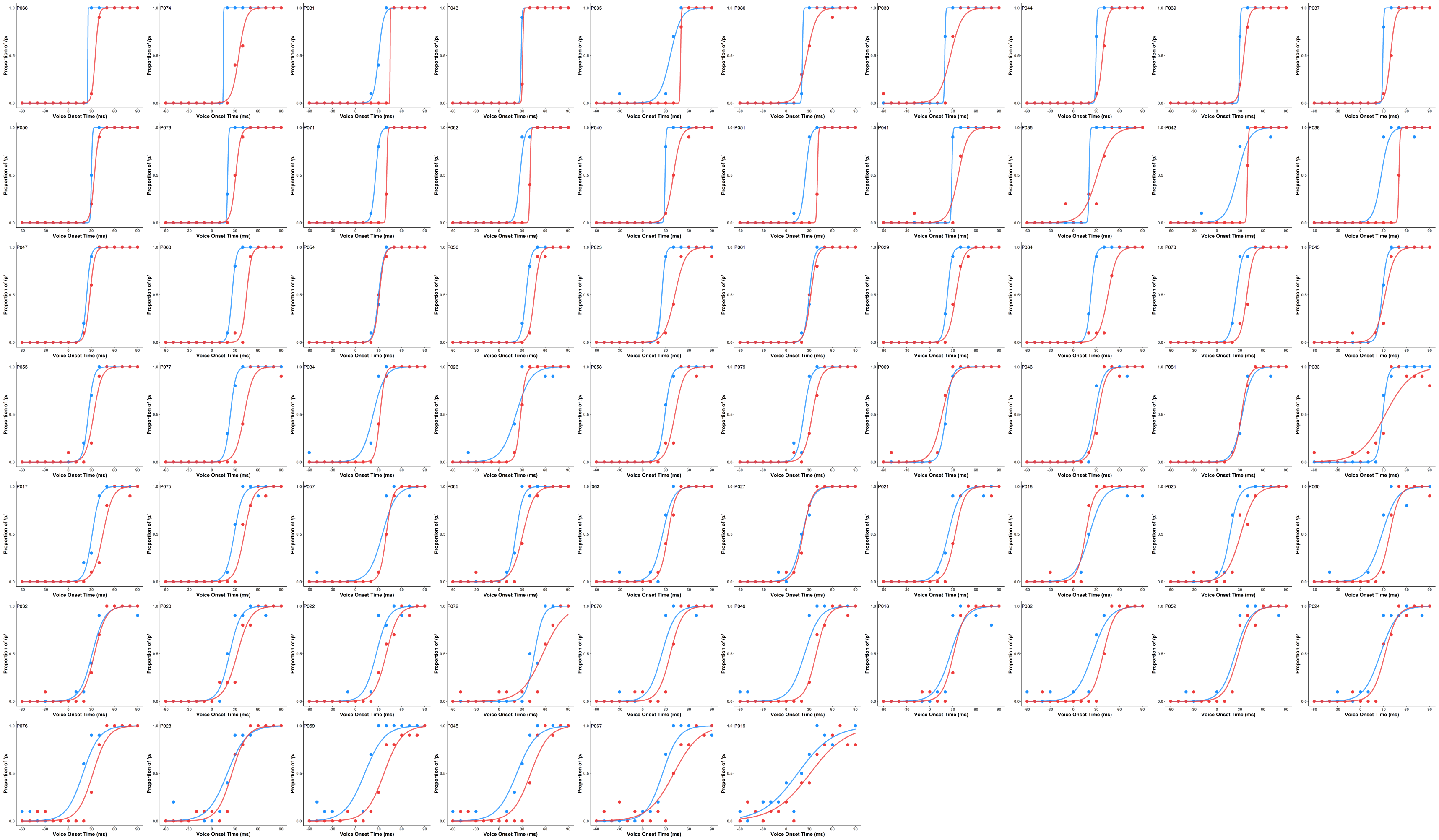

Psychometric curve fitting for each participant (main study: N = 66) ordered by mean slope value of the two phoneme identification tasks. Each language shown separately. English = blue; Mandarin = red.

Supplementary Table 5.

Fixed effects in linear mixed-effect model including fixed effects of test-language and *two* components derived from a Principal Component Analysis in the main study (N = 66), and random effects of participants (random intercept) for VOT boundary (Left) and slope value (Right).

	**GLMM for VOT boundary**				**GLMM for slope value**			
**Fixed effects**	**Estimate**	**Std. Error**	***t* value**	***p* value**	**Estimate**	**Std. Error**	***t* value**	***p* value**
(Intercept)	30.68	0.68	45.39	**<0.0001**	-1.37	0.10	-14.01	**<0.0001**
Test-language (Mandarin)	9.87	0.76	13.00	**<0.0001**	-0.24	0.16	-1.50	0.1386
PC1 (Bilingual balance)	0.23	0.68	0.34	0.732	-0.24	0.10	-2.43	**0.0177**
PC2 (Age of acquisition variance)	-0.38	0.68	-0.57	0.574	0.08	0.10	0.82	0.4127
Test-language:PC1	-0.01	0.76	-0.01	0.990	0.12	0.16	0.78	0.4407
**Random effects**								
Residual	19.02 (SD = 4.36)				0.82 (SD = 0.91)			
By-participant intercept	20.64 (SD = 4.54)				0.22 (SD = 0.47)			
N	66				66			
Observations	132				132			
AIC / BIC	853.5 / 873.7				391.1 / 411.3			
logLikelihood	-419.8				-188.6			

Larger t value in the GLMM for VOT boundary indicates a later threshold (ms) in the VOT continuum.

Smaller t value in the GLMM for slope value indicates a shallower slope of the phoneme identification curve.

Supplementary Table 6.

Power analysis for the main study (N = 66), based on the effect size of bilingual balance on slope.

**F tests** – Linear multiple regression: Fixed model, R^2 increase			
**Analysis:**	Post hoc: Compute achieved power		
**Input:**	Effect size f^2	=	0.0729614
	α err prob	=	0.05
	Total sample size	=	66
	Number of tested predictors	=	2
	Total number of predictors	=	2
**Output:**	Noncentrality parameter λ	=	4.8154524
	Critical F	=	3.1428085
	Numerator df	=	2
	Denominator df	=	63
	Power (1–β err prob)	=	0.4684602

Supplementary Table 7.

Reproducibility analysis for the main study (N = 66), based on the effect size of bilingual balance on slope.

**F tests** – Linear multiple regression: Fixed model, R^2 increase			
**Analysis:**	A priori: Compute required sample size		
**Input:**	Effect size f^2	=	0.0729614
	α err prob	=	0.05
	Power (1–β err prob)	=	0.95
	Number of tested predictors	=	2
	Total number of predictors	=	2
**Output:**	Noncentrality parameter λ	=	15.6867010
	Critical F	=	3.0384660
	Numerator df	=	2
	Denominator df	=	212
	Total sample size	=	215
	Actual power	=	0.9502873

Notes: To replicate the significant effects of bilingual balance on slope in the GLMM ($f^2 = 0.07$), and achieve power of 0.95 with alpha level of 0.05, a larger sample size of 215 would be required.
